# Indications and morbidity of tube thoracostomy performed for traumatic and non-traumatic free pleural effusions in a low-income setting

**DOI:** 10.11604/pamj.2014.18.256.3963

**Published:** 2014-07-27

**Authors:** Alain Chichom Mefire, Marcus Fokou, Louis Din Dika

**Affiliations:** 1 Regional Hospital Limbé and Faculty of Health Sciences, University of Buea, Yaoundé, Cameroon; 2 General and Reference Hospital, Yaoundé, Cameroon; 3 Regional Hospital Limbé, Limbé, Cameroon

**Keywords:** Tube thoracostomy, pleural effusion, traumatic, non-traumatic, indications, complications

## Abstract

**Introduction:**

Tube thoracostomy (TT) is widely used to resolve a number of pleural conditions. Few data are available on the complications of TT performed for non-traumatic conditions, especially in low income setting. The aim of this study is to analyse the indications and complications of TT performed for both traumatic and non-traumatic conditions of the chest in a low-income environment.

**Methods:**

This retrospective study conducted over a four years period in a the Regional Hospital, Limbe in South-West Cameroon analyses the rate and nature of complications after TT performed for both traumatic and non-traumatic conditions. Different factors related to complications are analysed.

**Results:**

We analysed 134 patients who had 186 chest tubes inserted. After placement, tubes were either connected to a water seal system (40%) or submitted to suction (60%). Most (91%) procedures were performed for a non-traumatic condition. Non-infectious pleural effusion in patients with HIV infection or pulmonary tuberculosis was the most common indication. Sixty six per-cents of procedures were carried out by a general surgeon. The complication rate was 19.35%. The most common complications included tube dislocation and pneumothorax. Most complications were solved by replacement of the tube. The nature of operator (general surgeon vs general practitioner) was a significant predictor of outcome (p < 0.01). No procedure related death was recorded.

**Conclusion:**

TT is a safe and efficient procedure to drain pleural collections of both traumatic and non-traumatic origins, even in low-income settings. The incidence of complications could be reduced by a better training of general practitioners on this procedure.

## Introduction

Tube thoracostomy (TT) is one of the most important modalities of treatment of pleural diseases of traumatic and non-traumatic origin. In thoracic injuries, this procedure is the most commonly used modality of treatment for hemothorax and pneumothorax [1–5]. The non-traumatic conditions associated with pleural effusion that require TT are numerous and vary according to geographical area, patient's epidemiological variables and local facilities for diagnosis and management [6–9]. In areas with high prevalence of HIV infection, it has been described to be associated with tuberculosis, *Pneumocystis carinii* infection, Kaposi's sarcoma and pneumonia [8, 9].

TT is known to be associated with a number of complications such as wrong placement, damage to lungs or mediastinal organs, bleeding or pain [3, 5, 10, 11, 12]. The rate of these complications is sometimes so high that the utility of TT is now being challenged, especially in the situation of chest injury [5, 13, 14]. Several attitudes have been proposed to reduce the complication rate, including radiological guidance [15, 16] or the introduction of smaller tubes with the idea of increasing patient's comfort and decreasing the rate of post-removal pneumothorax [17, 18]. Pleural effusions that require TT represent a special challenge in low-income environments because of the limitations in the availability and quality of equipment. The aim of this study is to analyse the indications and complications of chest tubes placed for both traumatic and non-traumatic conditions of the chest in a low-income environment.

## Methods

This retrospective observational study was conducted in the general surgery unit of Regional Hospital Limbe, a level III institution located in the South West region of Cameroon. The study covered a period of 4 years, from January 1st 2006 to December 31st 2009. During this period, every patient, irrespective of age and sex, who had a chest tube inserted and was admitted in a ward or observed in the hospital for a minimum period of 48 hours, was included in the study. We excluded all patients who left the ward or the hospital before 48 hours for various reasons (referral, disappearance, discharge or death). We also excluded all patients with an encysted pleural fluid collection. For each patient included, we used a standard data collection sheet to gather information regarding patient's background, indication for TT, previous thoracocentesis, location of chest tube insertion, technique of drainage, quality of operator, duration of drainage, complications recorded and outcome. Data were analysed using Epi-info 2000 and Chi-square test were used to compare proportions. P ≤ 0.05 was considered statistically significant.

## Results

A total of 203 chest tubes were inserted in 151 patients during the study period. 17 patients (11.26%) were excluded for the following reasons: discharge before 48 hours (n = 13) and death within less than 48 hours (n = 4). 134 patients who had 186 chest tubes inserted could finally be analysed. Two patients had a bilateral tube. 43 patients (32%) had TT performed more than once.

Their ages ranged from 2 to 67 (mean: 34.4 ± 7.6); the male to female ratio was 0.72. All patients were symptomatic at the time of placement of chest tube; chest pain and dyspnoea at various degrees were the most common symptoms.

Table 1 show that more than 91% of TT was performed for a non-traumatic condition. Most procedures were performed for a non-purulent pleural effusion (n = 93). Table 2 shows the associated co-morbidity in our patients: 59 patients with a pleural fluid effusion were smear positive tuberculosis patients under treatment, of which 38 (64.4%) had an associated HIV infection. All HIV infected patients had a CD4 count of less than 200. The presence of identified malignancy was rare (n = 3). All patients with a para-pneumonic infection were children below the age of 7.

**Table 1:** Indications for tube thoracostomy in 134 patients in Limbe, Cameroon

Indication	Number	Percentage
Post-traumatic haemotorax	6	4.47
Post-traumatric pneumothorax	1	0.74
Combined post-traumatic haemo-pneumothorax	5	3.73
Non-purulent Pleural effusion	93	69.42
Empyema thoracis	17	12.69
Para-pneumonic infection	6	4.47
Undetermined	6	4.47
**Total**	**134**	**100**

**Table 2:** Associated co-morbidity in patients undergoing tube thoracostomy in Limbe, Cameroon

Indication/Co-morbidity	HIV infection	Smear positive tuberculosis	Malignancy
Chest injury	0	0	0
Non-purulent Pleural effusion	38	59	2
Empyema thoracis	4	0	1
Para-pneumonic infection	1	0	0
Undetermined	2	1	0

Fifty seven patients had TT performed after a previous needle thoracocentesis performed. Of these patients, 18 (31.57%) had an associated pneumothorax on standard chest X-ray. The TT was performed by a surgeon 124 times (66.66%) and by a general practitioner in the remaining cases. It was performed in the operative room 153 times, the emergency department 21 times and in the ward 12 times.

There was an only one make-up chest tube available in our institution during the study period; this was a steel trocard tube (Kendall Argyle^®^, Tyco Healthcare). Almost all tubes were placed laterally in the 4th or 5th intercostal space. Once placed, the chest tube was connected to a water seal system 73 times (39.25%). All the remaining other chest tubes (60.75%) were submitted to a negative intra-thoracic pressure (suction) every day. All chest tubes were removed under continuous suction. No prophylactic antibiotic were administered.

The chest tube was left in place for 6 to 24 days with a mean of 9.3±3.8 days. All patients had at least one standard chest X-ray performed prior to TT. Follow-up was also based on regular chest X-rays. Another standard chest X-ray was systematically performed just before removal and immediately after.

We recorded 36 complications in 28 patients (19.35% complication rate). As shown on Table 3, chest tube dislocation and pneumothorax were the most common complication. The complication rate was significantly higher when the tube was placed by a general practitioner (69.44% vs 30.56%: p < 0.01). This rate was not significantly influenced by age, the mechanism of lesion (trauma vs non-trauma), the presence of co-morbidity and weather the TT was performed in the emergency department, the theatre or the ward. All three cases of wrong placement manifested as non functional chest tubes; they all were sub-cutaneous and identified as such with standard chest X-ray alone (antero-posterior and lateral views). The TT was then repeated successfully.

**Table 3:** Frequency of complications in patients undergoing tube thoracostomy in Limbe, Cameroon

Complications	Number of cases	Occurrence rate
Dislocation of tube	12	6.45%
Wrong placement (sub-cutaneous)	3	1.61%
Residual haemothorax	1	0.54%
Post-removal pneumothorax	6	3.22%
TT related Empyema thoracis	1	0.54%
Hypovolemic shock after massive drainage	1	0.54%
Drain site infection	5	2.69%
Pain at site of drain insertion	3	1.61%
Continuous drainage	2	1.07%
Non-functional (kinked?)	2	1.07%

One case of post-removal pneumothorax was symptomatic and required another TT. One case of non functional tube was treated with re-placement successfully; the second case failed and required referral for thoracotomy.

Thoracotomy was necessary for 5 patients (3.73%). These included two cases of failure of TT defined as continuous drainage after 20 days, one case of TT related empyema thoracis and the two cases of continuous drainage after removal of the drain. No procedure related mortality was recorded.

## Discussion

Tube thoracostomy (TT) is widely used to solve a variety of pleural conditions. The medical literature about the outcome of chest tube performed for traumatic chest conditions is rich [2–5, 13, 19–21], but data on TT for non-traumatic conditions are rather scarce [6, 10, 22, 23], especially in low-income settings [11]. We do not know of any combined analysis involving both traumatic and non-traumatic conditions. TT is known to be one of the most successful procedures in the management of pleural effusions of all types [3, 6]. But its use is limited in low-income setting because of its non feasibility, its cost and the availability of chest tubes. Needle thoracocentesis is then sometimes preferred.

HIV and tuberculous infections have been largely associated with increased risk of development of a pleural effusion [8, 9]. The relatively low mean age of our patients as compared to what is usually described [8, 11, 19] is probably explained by the fact that contrary to most studies, we associated injury cases which are known to be more frequent in young age [2, 5]. On the other hand, the rate of HIV infected cases is high, and this infection is known to be more frequent in young patients.

The rate of complications of chest tubes does not generally seem to be influenced by the nature of the lesion (trauma vs non-trauma) [3–5, 10, 21, 22]. In situations of chest injury, pneumothorax and haemothorax, often combined, are still the most frequent indications [2–5, 12, 13, 19–21].

In non-trauma related situations, tuberculosis and HIV infection seem to play a major role [8–11, 23, 24]. Valdés et al. found that tuberculous and cancerous pleural effusions were the most frequent [8]. According to Fartoukh and al., more than 50% of effusions are related to transsudates and non-infectious exsudates [14]. The scarcity of cancer related pleural effusions in our study is probably related to the absence of appropriate diagnostic facilities in our institution prohibiting a systematic search for the presence of neoplasm. The situation of empyema thoracis and para-pneumonic infections seem to represent a special challenge nowadays, especially in children [6, 23–26].

The utility of needle thoracocentesis have been recently reassessed in many studies, especially when this procedure is performed using a small bore catheter to reduce the risk of iatrogenic pneumothorax [6, 14, 17, 22, 27]. Though this procedure has shown to be efficient and safe especially in malignancy related pleural effusions [6, 27, 28], the incidence of iatrogenic pneumothorax could still be as high as 31% [17]. TT then seems to be a safer approach for large effusions in terms of having a lower complication rate. The overall complication rate of TT varies from 6% to 41.5% [3, 5, 10, 11, 19, 21, 22, 29]. Our complication rate is comparable to that recorded in many studies in well equipped environments [3, 4, 5, 19, 21]. In a setting with similar characteritics, Nwofor and al. recorded at least one complication in more than 41% of patients [11], half of which had a dislodgment of the tube (our most common complication).

Our choice to perform almost all our procedures in the lateral aspect of the chest is justified by the fact that there is no difference between anterior and lateral approaches in terms of functional malposition [30], though the incidence of interlobar malposition without functional consequences seems to be higher with the lateral approach [30]. We used both a water-seal system and suction in our study. In fact, both systems have shown equal efficacy on drainage and on removal of tube in preventing iatrogenic pneumothorax [6, 31].

The nature of the operator seems to be a clear predictor of outcome [10, 21, 22]. Khanzada and al described a complication rate as low as 5% when the procedure was performed by general surgeons [10]. There is then need to improve on the training of general practitioners on the procedure of TT as in low-income settings; they are the first line health care providers. Nurses may also have a major role to play and thus also deserve proper training in the management of chest tubes [32].

Emergency department and operative room seem to be safer than inward for TT [3, 19]. Positional complications such as malposition, failure and tube dislocation are generally the most frequent [5, 10–12]. This group of complication is probably underestimated in our study as they are sometimes difficult to diagnose with standard X-ray alone [33, 34]. But, they are significant only if associated with a failure (absence of drainage), as many locations such as interlobar might still be successful in draining the collection [30]. It is likely that the increasing use of radio guidance for insertion of chest tubes will significantly reduce the rate of positional complications [15, 16].

Our present policy of control X-ray will probably need to be revised. The immediate post-operative X-ray and the post-removal X-ray could be skipped without major risk in most patients [28, 35, 36]. This practice may need to be individualized [28]. Post-removal complications, mainly iatrogenic pneumothorax are also a frequent issue of concern [5]. Insertional and infective complications are rather rare [10, 37, 38].

The management of these complications still relies on conservative measures such as replacement of the tube, preferably under guidance [6, 15]. One of the exceptions is the situation of residual haemothorax after chest injury where the risk of empyema thoracis seems to be high [20]. The wise use of procedures such as open window thoracostomy and myoplasty which are feasible even in low-income settings, are likely to reduce the thoracostomy and thoracoscopy rate [6, 23, 24, 26], especially in critically ill patients [25]. We confirm that TT is not a life-threatening procedure when performed with minimum security measures [4, 10, 11, 13, 19, 22].

## Conclusion

When performed with caution, TT is a safe procedure to drain pleural collections of both traumatic and non-traumatic origins, even in low-income settings. It is more useful and cause fewer complications than needle thoracocentesis, especially in the management of large pleural effusions in the context of high prevalence of HIV and pulmonary tuberculosis. However, a significant number of complications could still occur of which positional complications are the most frequent. Their real incidence is likely to be higher if appropriate investigations such as CT scan are made available to help diagnose them. The overall complication rate in settings such as ours is likely to be reduced by a better training of general practitioners on the insertion of chest tubes, as it is probable that we will still need to rely on them for a significant number of procedures. It is also likely to be reduced by standardizing the management of chest tubes by the use of guidelines with predefined criteria [12, 39]. The rate of thoracotomies and thoracoscopies could also be successfully reduced by less invasive procedures such as thoracic window which is in our opinion a good option to be considered in the future by surgeons working in low-income setting, especially for critically ill patients who may not withstand a thoracotomy.
